# Recurrent tragal abscess associated with frequent in-ear headphone use: a case report

**DOI:** 10.1093/jscr/rjag612

**Published:** 2026-07-21

**Authors:** Mayar Alsaqr, Shahad Althubiti, Ahmed Alarfaj

**Affiliations:** Department of Otolaryngology—Head & Neck Surgery, King Saud University Medical City, King Saud University, King Abdullah Road, Al Wurud District, Riyadh 12372, Saudi Arabia; College of Medicine, Princess Nourah bint Abdulrahman University, Airport Road, Al Narjis District, Riyadh 84428, Saudi Arabia; Department of Otolaryngology—Head & Neck Surgery, King Saud University Medical City, King Saud University, King Abdullah Road, Al Wurud District, Riyadh 12372, Saudi Arabia

**Keywords:** tragal abscess, in-ear headphones, *Staphylococcus aureus*otitis externa, case report

## Abstract

Localized infections of the auricle and tragus are uncommon. Recurrent disease should lead clinicians to ask about possible risk factors and contributing factors. A 39-year-old medically and surgically free man presented with a 3-day history of painful right tragal swelling preceded by localized discharge. He denied fever and trauma. He had two earlier similar episodes affecting both ears at different times, both resolving spontaneously. He reported frequent prolonged use of in-ear headphones, with symptoms usually following heavy in-ear headphone use. Incision and drainage were done, and pus culture grew *Staphylococcus aureus*. Initial clindamycin dosing was incorrect, so treatment was subtherapeutic. He was then switched to trimethoprim-sulfamethoxazole along with topical mupirocin with clear improvement. At follow-up, pain and discharge had resolved and the swelling was smaller. This case suggests that prolonged in-ear headphone use may be a contributing factor in recurrent tragal abscess.

## Introduction

Tragal and other localized auricular infections are uncommon, and recurrence should prompt clinicians to look beyond a single episode. Damage to the external-ear skin barrier may permit bacterial entry, and common organisms include *Staphylococcus aureus* and *Pseudomonas aeruginosa* [1].

In this setting, devices worn in or around the ear deserve attention. Occlusive ear equipment can increase bacterial counts in the external auditory canal, suggesting that covered warm or moist ear spaces may support bacterial growth [2]. *Staphylococcus aureus* external otitis has also been reported after airline headset use [3].

Reports related to earphones are also clinically useful. External-ear problems related to excessive earphone use have been reported in children, including bilateral otitis externa and a small conchal pressure ulcer after sleeping with earbuds in place [4]. Against this background, this case presents a healthy adult man with recurrent tragal abscess and a repeated temporal association with prolonged in-ear headphone use.

## Case report

A 39-year-old medically and surgically free man presented with 3 days of right tragal swelling and tenderness preceded by localized discharge. He denied fever, trauma, recent ear procedure or known injury. He reported two similar episodes affecting both tragal sites at different times, both resolving spontaneously as self-draining abscesses.

On further history, he reported frequent and prolonged use of in-ear headphones with no history of sharing headphones with others. He used them for long periods during the day. He noticed that symptoms usually started after heavy device use.

**Figure 1:**
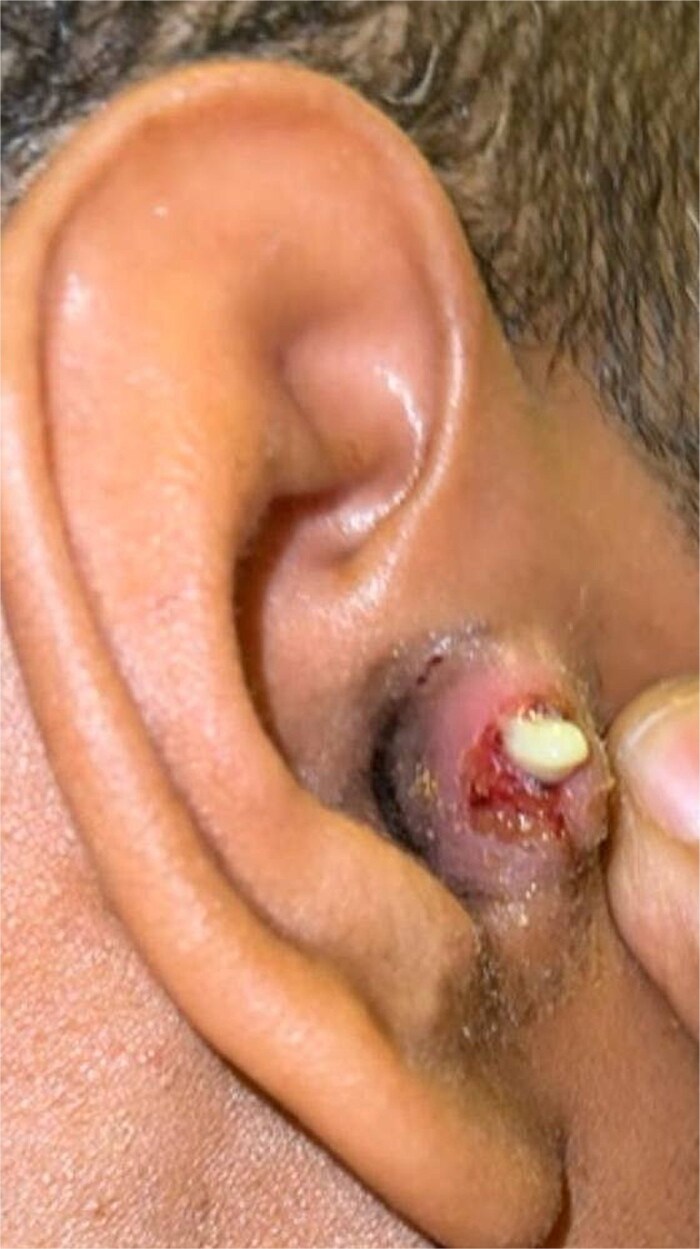
Clinical photograph at initial presentation showing right tragal swelling with spontaneous purulent drainage from a small surface opening.

On examination, there was localized swelling and tenderness over the right tragus. The surrounding auricle was otherwise not inflamed. There were no systemic symptoms. A clinical photograph taken at initial presentation showed a tense tragal swelling with spontaneous purulent drainage from a small surface opening (Fig. 1).

The clinical diagnosis was a localized right tragal abscess. Incision and drainage were performed. Purulent material was sent for culture. The patient was initially prescribed clindamycin, but he was taking a subtherapeutic dose.

The culture grew *S. aureus*. The antibiotic was changed to trimethoprim-sulfamethoxazole for a total of 7 days along with topical mupirocin. The patient was counseled on correct antibiotic dosing, device cleaning, avoiding device sharing and reducing prolonged in-ear headphone use, especially during active irritation or discharge.

**Figure 2:**
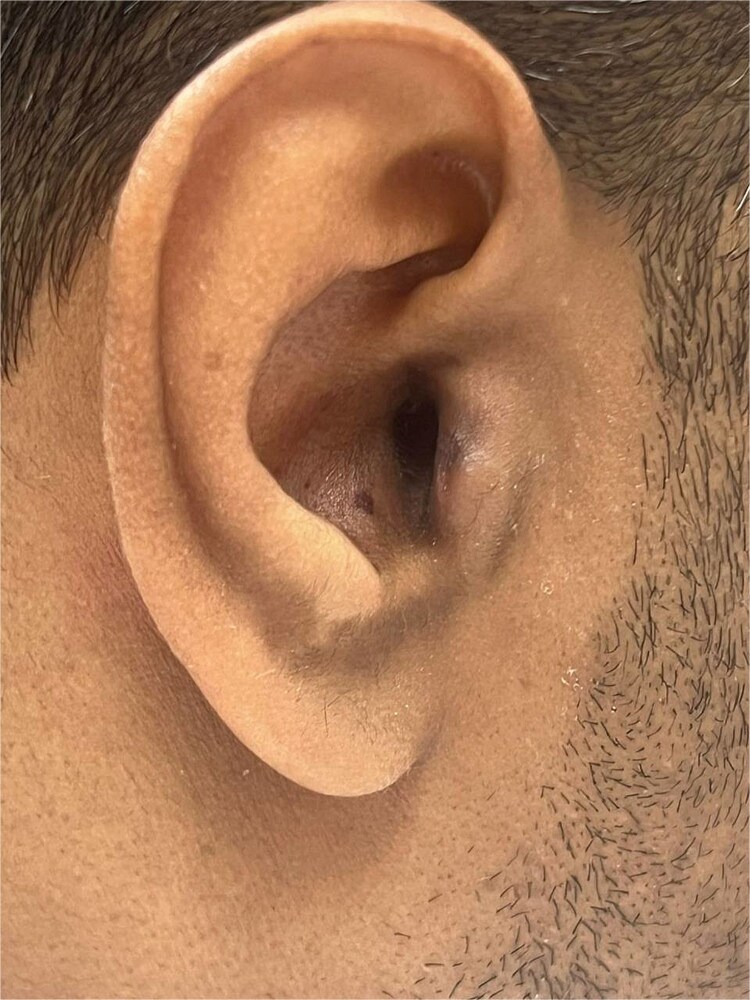
Post-treatment clinical photograph of the right external ear at follow-up showing resolution of the acute swelling and erythema with mild hyperpigmentation and no visible discharge.

At follow-up, the pain and discharge had resolved. Examination showed a smaller right tragal swelling. It was non-tender and there was no expressible discharge. Final follow-up clinical photograph showed resolution of the earlier swelling and erythema, with mild hyperpigmentation and no discharge (Fig. 2).

## Discussion

This case involved a culture-proven *S. aureus* tragal abscess with repeated symptoms after prolonged in-ear headphone use. Microbial counts on airline headset devices have been shown to rise after 1 h of wear, with staphylococci among the recovered organisms [5]. Frequent or shared earphone use has also been associated with increased bacterial growth in ears and on earphones [6]. However, this patient had no history of sharing headphones, and he reported frequent earphone use with the same timing of this episode and the previous two episodes of tragal abscess. El-Sakhawy *et al.* reported microbial hazards in earset users and found links between bacterial isolates, device type, sharing and ear symptoms [7].

Furthermore, other studies support asking about earphone habits. A Central India study found higher rates of impacted wax, otomycosis and otitis externa among earphone users than non-users [8]. A web-based survey found otitis externa symptoms or diagnosis in 21% of recent earphone users [9]. Saudi data linked long-term headphone use with ear-related complaints [10], and a recent cross-sectional study identified prolonged wear and sharing as concerning habits while culturing organisms from earphone samples [11]. Taken together, the current literature links earphone use to device contamination, bacterial growth, otitis externa and ear symptoms. While no reported cases have linked the possibility of in-ear headphone use with recurrent tragal abscess formation, our case describes recurrent tragal abscess following prolonged in-ear headphone use as noted by the patient, with the most recent episode being culture-proven *S. aureus* infection in a medically free adult.

Hearing aid studies show that devices placed in the external canal can change local flora [12]. Recurrent *S. aureus* external otitis related to stethoscope earpieces resolved after cleaning, supporting advice on cleaning personal ear devices [13]. Zia *et al.* studied ear infection among headphone users and found that headphone use was significantly associated with ear infection, itching, and hearing-related complaints [14]. Our case supports this association but adds a more specific finding because the patient developed recurrent localized tragal abscesses after prolonged in-ear headphone use in a healthy, medically and surgically free adult.

Finally, Mazlan *et al.* did not find a clear link between prolonged headphone use and external ear infection [15]. In contrast, our case suggests a possible association because three recurrent tragal abscess episodes were temporally related to prolonged in-ear headphone use. Management included incision and drainage, culture-guided oral therapy, and correction of subtherapeutic dosing. The patient improved after 7 days of trimethoprim-sulfamethoxazole with topical mupirocin. As additional precautions, he was advised on correct antibiotic dosing, device cleaning, avoiding device sharing, and reducing prolonged in-ear headphone use during active irritation or discharge.

In conclusion, this case suggests that frequent in-ear headphone use may contribute to recurrent tragal abscess through local friction, occlusion and moisture retention may allow repeated bacterial exposure. Although current literature reports bacterial colonization, otitis externa and device contamination, reports of tragal or localized external-ear abscess related to headphone use remain limited. Therefore, our case is clinically important because it describes a healthy adult with recurrent culture-proven *S. aureus* tragal abscess after prolonged in-ear headphone use. Clinicians should ask about headphone habits and advise patients to clean devise regularly, avoid sharing, reduce prolonged use during irritation or discharge and complete antibiotics course as prescribed.

## Data Availability

All data supporting this case report are included within the article.
